# A high-frequency single nucleotide polymorphism in the MtrB sensor kinase in clinical strains of *Mycobacterium tuberculosis* alters its biochemical and physiological properties

**DOI:** 10.1371/journal.pone.0256664

**Published:** 2021-09-16

**Authors:** Uchenna Watson Waturuocha, Athira P. J., Krishna Kumar Singh, Vandana Malhotra, M. S. Krishna, Deepak Kumar Saini

**Affiliations:** 1 Department of Studies in Zoology, Manasagangotri, University of Mysore, Mysore, India; 2 Department of Molecular Reproduction, Development and Genetics, Indian Institute of Science, Bangalore, India; 3 Department of Biochemistry, Sri Venkateswara College, University of Delhi, Delhi, India; 4 Center for Biosystems Science and Engineering, Indian Institute of Science, Bangalore, India; King Faisal Specialist Hospital and Research Center, SAUDI ARABIA

## Abstract

The DNA polymorphisms found in clinical strains of *Mycobacterium tuberculosis* drive altered physiology, virulence, and pathogenesis in them. Although the lineages of these clinical strains can be traced back to common ancestor/s, there exists a plethora of difference between them, compared to those that have evolved in the laboratory. We identify a mutation present in ~80% of clinical strains, which maps in the HATPase domain of the sensor kinase MtrB and alters kinase and phosphatase activities, and affects its physiological role. The changes conferred by the mutation were probed by *in-vitro* biochemical assays which revealed changes in signaling properties of the sensor kinase. These changes also affect bacterial cell division rates, size and membrane properties. The study highlights the impact of DNA polymorphisms on the pathophysiology of clinical strains and provides insights into underlying mechanisms that drive signal transduction in pathogenic bacteria.

## Introduction

The global burden of tuberculosis continues to increase annually, with the emergence of multi, extensive and completely drug-resistant strains of *Mycobacterium tuberculosis* (*M*. *tuberculosis*). The ability of mycobacteria to undergo dormancy and reactivation makes it a difficult pathogen to tackle. The current knowledge about the Mycobacterial strains obtained from large-scale sequencing projects has revealed genomic diversity among clinical strains. The underlying cause of this diversity is the presence of transposable elements and nucleotide polymorphisms that result in various phenotypic variations such as drug resistance, virulence, growth and pathogenicity [1–3]. Polymorphisms, including single nucleotide substitutions, deletions, or duplications, have been shown to drive the evolution of strains and lineages of *M*. *tuberculosis* [4] and are responsible for the differences in growth rates [5], membrane composition [6], proteome [7], along with variations in the expression of genes of different families, including those regulating host-pathogen interactions [8]. Although the number and degree of variations within and across strains vary, they have been proved to be major drivers of divergence among various mycobacterial strains [9]. However, studies of mutations in signaling proteins that govern the bacterium’s adaptation and survival in its ever-changing hostile environment have been minimal, accounting for the poor understanding of their impact in clinical strains.

The adaptation of mycobacteria to its environment both outside and within its host is governed by two types of signaling systems: the Two Component Systems (TCS) and the Serine Threonine Protein Kinases (STPK). TCSs, as the name, suggests consist of two components, one involved in sensing and the other in responding [10]. The sensory protein is a kinase (SK), which responds to external stimuli and undergoes autophosphorylation at a conserved Histidine (His) residue. The response regulator (RR) protein receives the phosphate group from the SK by a phosphotransfer event leading to phosphorylation of a conserved Aspartate (Asp) residue that alters its DNA/ RNA binding ability, thereby changing the transcription profile of the bacterium in response to the environmental cues [11–13]. *M*. *tuberculosis* has 12 pairs of two component systems [14, 15], which regulate several physiological processes and pathogenesis in the bacteria.

The *mtrAB* TCS system (Rv3245c-Rv3246c), present in all actinobacteria, was one of the first TCS characterized in *M*. *tuberculosis* [15, 16]. It is now known that the RR *mtrA* is essential, unlike the SK *mtrB* [14]. This TCS regulates cell division and replication, cell wall synthesis, and cell morphology [16–18]. Studies with an *mtrB* deletion mutant revealed that it is also involved in biofilm formation and aids in withstanding hypoxia and acid stress by interacting with- and regulating other two component systems [14, 19]. The expression of the *mtrA* gene and three other RRs is also known to be higher in Multi-Drug Resistant (MDR) strains of *M*. *tuberculosis* [20], hinting towards possible roles in the altered drug-resistance properties of the clinical strains.

While efforts are ongoing to consolidate changes or variations between laboratory and clinical strains at the genomic and proteomic levels, individual polymorphisms are seldom characterized to assess their phenotypic impact on the bacterium. In the present study, we characterize a specific Single Nucleotide Polymorphisim (SNP) in MtrB sensor kinase found in clinical strains of *M*. *tuberculosis*, which was found to alter the kinase and phosphatase property of the protein *in vitro*. We demonstrate that such a change can enhance the DNA binding ability of MtrA RR, affecting downstream regulation of gene expression resulting in variations in growth rate, cell size and, cell membrane properties of the strain carrying the MtrB variant.

## Materials and methods

Chemicals, media, biochemicals, and protein reagents were obtained from Merck (USA), Restriction Enzymes were from Thermo-Fisher (USA). The protein marker was obtained from Abcam (UK). Cloning and qRT primers were synthesized by Bioserve (India). Radioactive γ^32^P ATP (>4000 Ci/mmol) was from BRIT-Jonaki (Hyderabad, India). Middlebrook 7H9 and 7H11 were obtained from BD (USA), Trizol from TaKaRa (Japan). 0.1mm zirconia beads from Biospec Products Inc. (USA). iScript cDNA synthesis kit from BioRad (USA). DyNAmo Color Flash SYBR Green qPCR Kit from Thermo Fisher (USA).

### Insilco sequence analysis

SNPs in the *mtrAB* two-component system region were examined from the GMTV database (Genome Wide *M*. *tuberculosis* Variation database) (http://mtb.dobzhanskycenter.org) [21], and comparative sequence analysis was performed using H37Rv (NC_000962) as a reference wild type strain. A variation at the position from ***A****tg* to ***C****tg* at the 1549 nucleotide of the *mtrB* gene was identified and was labeled *mtrB’* (M517L). The sequences were analyzed and aligned using T-COFFEE and CLUSTALW [22] using the *mtrB* gene sequence with the H37Rv sequence as the template strain.

### Recombinant plasmid construction and generation of bacterial strains

The overexpression of proteins was carried out in BL21 Arctic Express^TM^ (Agilent Technologies, USA) *E*. *coli* strains after cloning in DH10β *E*. *coli* strains and grown in LB medium with 100 μg/ml ampicillin or 50 μg/ml gentamycin, respectively. A list of primers used for PCR and cloning is listed in S1 Table in S2 File. The recombinant plasmids used for protein overexpression are previously reported [23]. For the generation of the mycobacterial expression plasmid containing the *mtrAB* operon, the nucleotide region of the sensor kinase, *mtrB* was PCR amplified from H37Rv genomic DNA using specific primers and cloned in pMV261 vector between the EcoRI and HindIII restriction sites, all the recombinant constructs generated were verified by DNA sequencing.

### Expression and purification of recombinant proteins

The expression and purification of recombinant proteins (both wild type and mutant) were carried out as previously reported [24].

### Phosphorylation analysis

The autophosphorylation assays were performed as previously described [23]. In brief, 50 pmoles of the purified SKs (MtrB WT and MtrB’ M517L) were autophosphorylated in kinase buffer (50 mM Tris-Cl (pH 8.0), 50 mM KCl, 20 mM MgCl_2_), 100 μM ATP and 2 μCi of γ^32^P-ATP (>4000 Ci/mmol, BRIT, India) for 60 min or at indicated times (during time-course experiments) at 30°C. The reaction was terminated using a 1× SDS-PAGE buffer and resolved on a 12.5% SDS-PAGE gel. For the phosphotransfer assays, 150 pmol, the cognate RR, MtrA, diluted in kinase buffer, was incubated for different times (as indicated) at 30°C with the phosphorylated SK (MtrB WT/MtrB’ M517L~P) (phosphorylated for 60 min). The addition of 1xSDS-PAGE buffer terminated the reaction, and the samples were resolved on a 12.5% SDS-PAGE gel. The gels were washed and exposed to a phosphor screen (Fujifilm, Japan), imaged with the Typhoon phosphorimager (GE Healthcare, USA). The Microsoft image editing tool was used to adjust the brightness and contrast of the images obtained, and ImageJ was used to perform a quantitative densitometric analysis of the autoradiograms. For quantification, the first time point’s signal was considered 100%, and relative levels of phosphorylation over time for the wild type and mutant SKs were determined. In the phosphotransfer assays, the first time point of the RR~P generated by the wild type MtrB is considered 100%, and relative levels of phosphorylation over time were calculated. Statistical analysis, significance (p values) was calculated with reference to SK~P levels in the autophosphorylation reaction.

### Dephosphorylation analysis

The dephosphorylation assay was performed as previously reported [25] with modifications as specified below. In brief, 50 pmoles of the purified WT MtrB were autophosphorylated in kinase buffer using γ^32^P-ATP for 60 min at 30°C, as described above. The reaction mix was incubated with Magnetic Ni-NTA beads for 15 mins, and the excess ATP was removed. Labelled MtrB WT~P was eluted from the beads using elution buffer (25 mM Tris.Cl pH 8.0, 500 mM NaCl, 250 mM imidazole, 10% glycerol). The purified and labelled protein was incubated with tagless MtrA (generated by rTEV digestion to remove the 6× Histidine tag) for 30 mins to generate MtrA~P. This was followed by eliminating MtrB from the reaction using magnetic Ni-NTA beads (as above after 10x dilution). Labelled MtrA~P was incubated with equal amounts of WT or mutant MtrB. The reactions were terminated at the indicated time points with 1x SDS-PAGE buffer and resolved on a 12% SDS-PAGE gel followed by imaging as described above. For quantification, the signal from MtrA~P alone was considered 100%, and relative levels of phosphorylated MtrA~P at various time points were determined.

### TLC to estimate ATP hydrolysis and PI release

For Thin Layer Chromatography, the phosphotransfer assay, as described above, was performed at a 10 μl volume and terminated using 50mM EDTA at the indicated time points. The amounts of labeled iP released from the SK~P was determined by spotting 1μl of the sample on polyethyleneimine-cellulose plates and resolved using 2M HCOOH, 2M LiCl (2:1) as the mobile phase; the dried plates were exposed to a phosphor screen (Fujifilm, Japan) and scanned with Typhoon phosphorimager (GE Healthcare, USA). A quantitative comparison for the amount of ^32^iP released was made by normalizing the later time points in the presence of the RR to the iP generated by the SK alone.

### Electrophoretic Mobility Shift Assay (EMSA)

A 526 bp DNA fragment corresponding to the *oriC* region was PCR-amplified from *Mycobacterium tuberculosis* H37Rv genomic DNA template using specific primers (S2 File). The PCR products were purified and end-labeled with γ^32^P-phosphate using T4 polynucleotide kinase (Thermo Scientific, USA) as per the manufacturer’s protocol. The labeled fragments were purified and used for EMSA as described previously [26], by incubating with the indicated amount of MtrA protein for 45 min at 25°C in the binding buffer (25 mM Tris.Cl pH 8.0, 20 mM KCl, 6 mM MgCl_2_, 0.10 mg/ml BSA, 0.5% glycerol, 1 mM DTT, 0.5 mM EDTA and 1 μg poly dI.dC). SK proteins were autophosphorylated with 5 mM ATP and incubated with MtrA to obtain MtrA~P. The reaction mixtures were resolved on a 4% native polyacrylamide gel (29.5:0.5), pre-equilibrated for 1–2 h at 80 V in 0.5× Tris-borate EDTA buffer at 4°C. Electrophoresis was performed at 4°C at 80 V for 2–3 h. The DNA-protein complexes were visualized by phosphorimaging as described above.

### Strains and growth conditions

The *Mycobacterium tuberculosis* strain H37Ra, obtained from Prof. Amit Singh, Departement of Microbiology, Indian Institute of Science, Bangalore, was grown in liquid broth of Middlebrook 7H9 medium containing 0.05% Tween80 and 10% OADC (containing BSA, dextrose, sodium oleate, and catalase). The overexpression strains were grown in the same medium containing 25μg/ml of Kanamycin.

### Mycobacterial growth assays

The *Mycobacterium tuberculosis* H37Ra strains containing the pMV261 plasmid alone (vector control), pMV261 containing *mtrB* WT gene sequence (*mtrB* WT), and pMV261 containing *mtrB* mutant gene (*mtrB’ M517L*), were grown to mid-log phase in Middlebrook 7H9 medium. The precultures were pelleted at 4500 rpm for 10 min and washed twice with 1 x PBS, then diluted to an OD_600_ of 0.05 in Middlebrook 7H9 medium with 10% OADC and 0.05% tween80. OD readings were measured at 600 nm absorbance for 12 days.

### Transmission electron microscopy

The samples for TEM imaging were prepared and examined, as described earlier [27]. Briefly, bacterial cultures were fixed with 4% paraformaldehyde. These were then observed by electron microscopy after negative staining. Approximately one drop of the fixed sample from each culture was placed onto a 300-mesh carbon formvar covered copper grid each, and the excess sample was removed using blotting paper. The samples were negatively stained using 1% uranyl acetate in 70% methanol (v/v). The morphology of the bacilli was examined using T12 BioTwin Transmission Electron Microscope (FEI, Tecnai, USA).

### Atomic force microscopy and cell wall stiffness measurements

The cultures used for AFM were grown to mid-log phase in Middlebrook 7H9 medium containing 10% OADC and prepared as previously described [28]. Samples were centrifuged at 4500 rpm for 5 min, followed by 3 washes with sterile milliQ water to remove residual media components. The OD_600_ was set to 0.1, and 100μl of the sample was added onto poly-L-lysine coated coverslips. The samples were then air-dried at room temperature for an hour, and the non-adherent bacteria were washed off with multiple washes of sterile milliQ water; the coverslips were air-dried for an hour again and mounted onto the magnetic stub of the AFM stage using double-sided carbon tape. All the AFM measurements were performed using the NX-10 Atomic Force Microscope (Park Systems, South Korea) in the non-contact mode. A silicon probe tip with a high-force constant was used at a resonating frequency of 300kHz. The cantilever had a pyramid-shaped tip with a radius less than 10μm, width of 35μm, length of 125μm, and thickness of 4.5μm.

### Statistical analysis

Statistical tests were performed using one-way ANOVA for phosphorylation assays, qRT-PCR, TEM, AFM, and MIC experiments. The data was represented as mean ± SE and p-values indicate * < 0.05, ** < 0.01, *** < 0.001, and **** < 0.0001.

## Results

### Identification of SNPs in the sensor kinase MtrB

**Fig 1:**
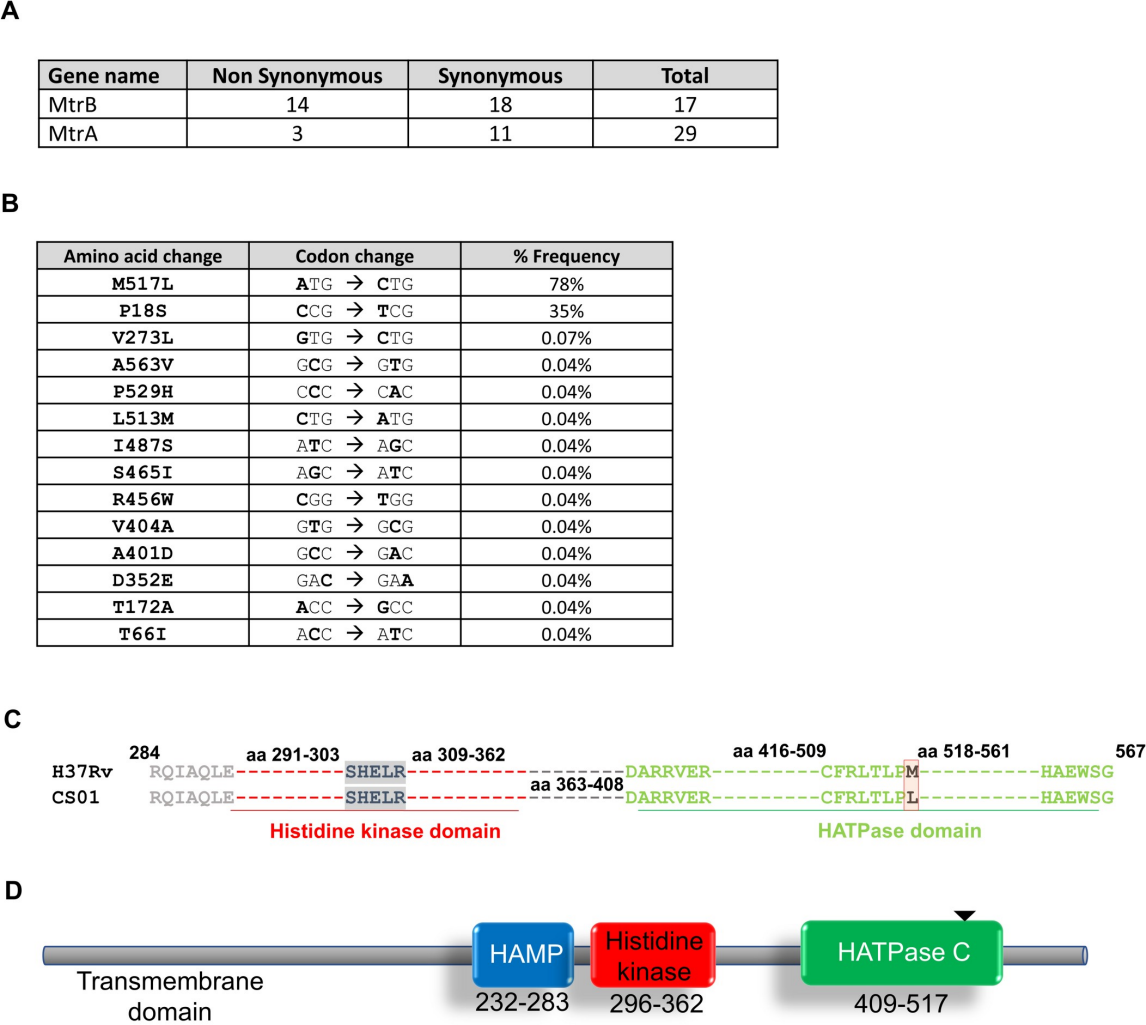
Sequence analysis to identify the SNP in MtrB sensor kinase. **A.** Total SNPs found in the DNA sequence of the *mtrAB* TCS in the 2501 strains of *M*. *tuberculosis* (sequences available in GMTV database). **B.** A list of nonsynonymous SNPs found in the MtrB protein with the codon changes and their percentage frequencies. **C.** Sequence alignment of the protein showing the histidine kinase and HATPase domain of the sensor kinase protein MtrB, using H37Rv as the reference strain. The alignment of sequence from H37Rv and one representative strain from GMTV carrying the mutation is shown. SHELR box in the kinase domain is marked in bold and shaded grey with the mutation from Methionine to Leucine at the 517^th^ residue marked in bold; CS1 is the number assigned to the representative clinical strain used for alignment. **D.** Pfam based predicted structure of the sensor kinase protein MtrB, highlighting three distinct domains: the HAMP domain, the Histidine Kinase (HK) domain, and the HATPase domain. The mutation at the 517^th^ position, which lies in the HATPase domain, is marked by the black arrow.

Comparative sequence analysis of the SNPs (single nucleotide polymorphism) found in the GMTV database (Genome-wide Mycobacterium tuberculosis Variation) with *M*. *tuberculosis* H37Rv laboratory strain sequence (NCBI reference sequence no. NC_000962) as a reference identified various non-synonymous and synonymous changes in the MtrAB proteins (Fig 1A). Though the number of synonymous SNPs outnumbered the non-synonymous ones, the frequency of a few of the non-synonymous SNPs was considerably high (Fig 1B). The SNP at position 1549 in the *mtrB* gene generated an amino acid variant of MtrB protein with Methionine to Leucine substitution at the 517 position. This SNP had the highest frequency of occurrence, 78% (Table 1 and Fig 1B) and mapped in the HATPase domain (Fig 1C and 1D), thus making this SNP an intriguing candidate to characterize. This mutation is also present in *M*. *leprae*, *M*. *marinum*, JAL2287, Harlem, and *M*. *tuberculosis* Erdman (Table 1); however, it was found to be absent in H37Ra, the avirulent counterpart of H37Rv [29], allowing us to use H37Ra as a host strain to examine the impact of this mutation.

**Table 1:** Table shows the frequency of the mutation occurring in different strains of *Mycobacterium tuberculosis* and prevalence in the GMTV database.

Gene	His residue	Mutation in reference to H37Rv	Lab strains containing this mutation	Frequency in GMTV database*
MtrB	305	M517L	*CDC1551*, *Erdman strain*, *Harlem strain*, *JAL2287*	1951/2501

*The GMTV database consists of 2501 clinical genome sequences that have been aligned for mutations in different regions of the bacteria.

### The M517L variant of MtrB sensor kinase has lower catalytic activity

**Fig 2:**
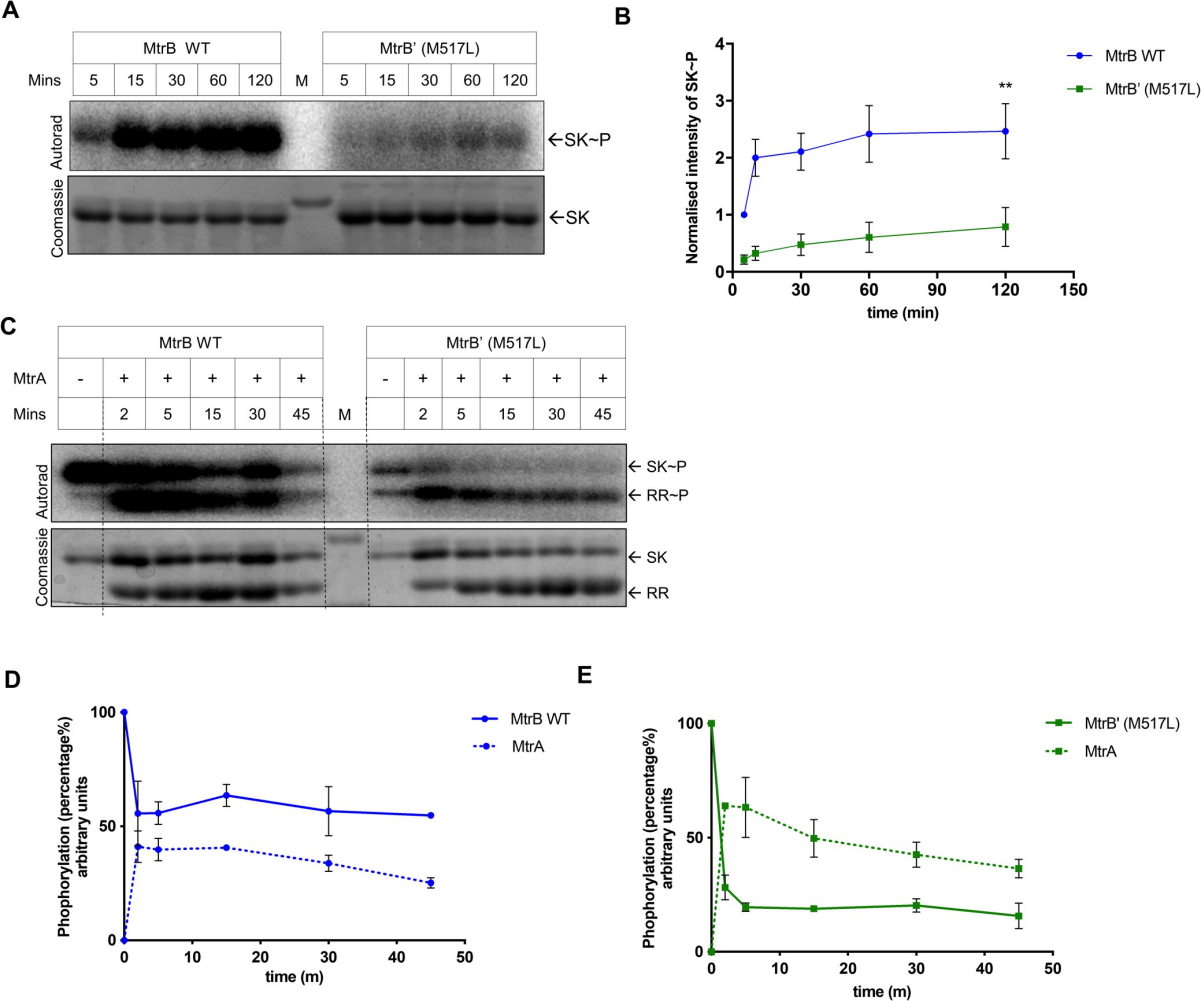
Assessment of the autophosphorylation and autokinase activity of the sensor kinase MtrB carrying M517L mutation. **A.** Autophosphorylation time-course analysis for the mutated and wild-type MtrB SK protein. M, Marker. **B.** Quantitative measurement of phosphorylation of the WT and mutant MtrB proteins at various time points (as shown in Fig 2A). The signal recorded for both proteins at the first time point was taken as 1 and the later time points were normalized to it (n = 3). **C.** Phosphotransfer time course to analyze the effect of the mutation on the phosphotransfer efficiency to the RR MtrA. The mutated or the wild-type MtrB protein were incubated with the RR MtrA (after autophosphorylation) in the phosphotransfer assay. M, marker. **D and E.** Quantitation of phosphorylated SK MtrB~P and RR MtrA~P protein at various time points (generated through wild type or and mutant MtrB as shown in Fig 2C). The signal recorded by the phosphotransfer of the SK at the first time point was taken as 100%, and the subsequent time points were normalized to it (n = 3) (p values; *≤ 0.05, **≤ 0.01).

Given that the mutation maps in the C-terminal catalytic domain of the MtrB sensor kinase protein, we decided to examine the effect of the mutation on the catalytic activities of the protein. We also modelled the 3D structure using PHYRE [30] and compared the structures for folding differences between the WT and mutant (M517L) proteins (S1A Fig in S1 File). However, we failed to record differences between the WT and mutant (M517L). To assess the effect of the amino acid substitution on the biochemical activity, we introduced the mutation into the gene sequence coding for the C-terminal cytosolic domain of the sensor kinase, cloned in the pProEx expression vector [23]. The protein was purified for *in vitro* activity analysis (the MtrB M517L mutant protein is hereafter mentioned as MtrB’). The C-terminal domain of SK proteins has previously been shown to be biochemically active [23, 31]. Post purification, CD spectroscopy confirmed the absence of structural differences between wild-type and mutant proteins (S1B Fig in S1 File). We then analyzed the kinase activities of the purified proteins by *in vitro* phosphorylation assays using γ^32^P-ATP as a substrate. We observed that the mutant protein’s autophosphorylation efficiency was significantly lower than the wild-type MtrB protein (Fig 2A and 2B). Next, we analyzed the effect of the mutation on the phosphotransfer efficiency to the cognate RR protein MtrA. In this reaction, the ^32^P labelled SK is incubated with the RR, and the appearance of ^32^P labelled RR is monitored. We observed that, though the mutant protein had poor autokinase efficiency (Fig 2C–2E), it generated higher levels of phosphorylated RR MtrA~P, which were also sustained over time, unlike in the reaction when it is generated through the wild type MtrB protein (Fig 2D). The observations pointed towards two previously unreported aspects of the MtrB sensor kinase, first, the presence of inherent phosphatase activity in the SK protein, and second, poor dephosphorylation ability of the mutant SK towards RR MtrA. Overall, we observed the mutation affected all the core catalytic activities viz. kinase, phosphotransferase, and phosphatase of the MtrB sensor kinase protein.

### Mutation in the sensor kinase alters its phosphatase activity

**Fig 3:**
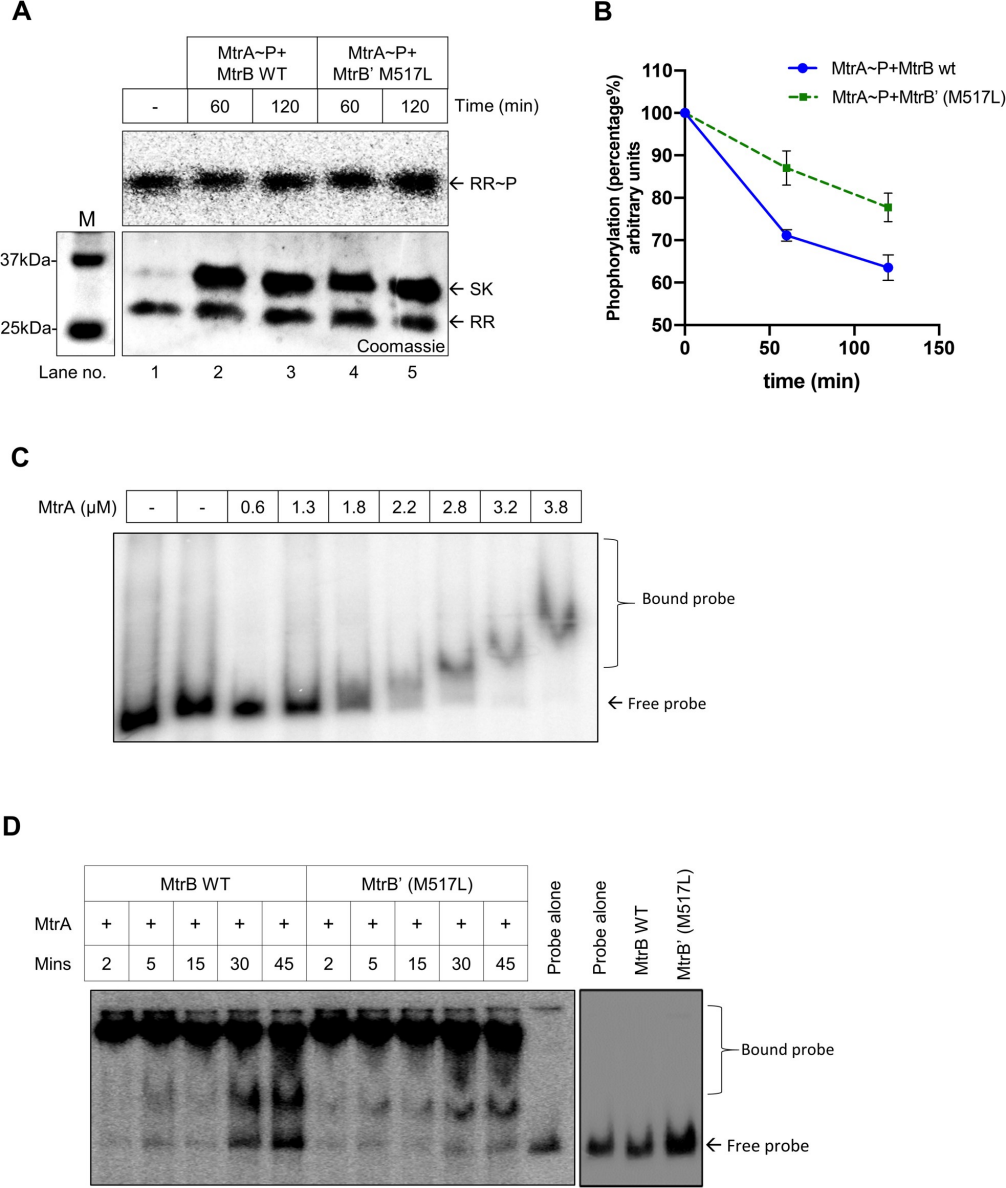
Effect of the mutation on the phosphatase activity of the SK on RR~P and its DNA binding ability. **A.** Dephosphorylation analysis of MtrA~P through wild type or mutant MrB. MtrA~P was generated as described in the Methods section and incubated with the wild type or mutant MtrB for the indicated time points. M, marker. **B.** Quantitative measurement of the amount of MtrA~P remaining at the indicated time points is shown in Fig 3A. The signal recorded with MtrA~P in the absence of SK was taken as 100, and the other time points were normalized to it to calculate the percentage of phosphorylated RR (n = 3). **C.** Titration of MtrA RR protein concentration to determine the minimal concentration of unphosphorylated MtrA which can bind to the *oriC*. Labeled DNA were incubated with increasing concentration of MtrA protein (as indicated) and tested for mobility shift of the probe by EMSA. The RR KdpE was used as a negative control to evaluate nonspecific binding (lane 2). **D.** A comparative analysis of MtrA binding to *oriC* DNA as a function of phosphorylation through wild-type or mutant MtrB’ SK proteins used at different concentrations. For all experiments, n = 3 biological replicates and a representative image is shown.

The phosphatase activity of SK forms a critical regulatory node in a typical TCS cascade [12] by facilitating the resetting of TCS signaling. It has been proposed that SKs mediate dephosphorylation of the cognate RRs by removing the phosphoryl group from the RR, and in the process, generate free inorganic phosphate [12]. Given that the phosphotransfer experiment suggests an altered phosphatase activity of mutant MtrB, we examined the dephosphorylation rate of RR MtrA~P, in the presence of WT and mutant SKs (Fig 3A). The signal intensities from MtrA~P in the presence of MtrB at different time points when normalized to the MtrA~P alone (Fig 3A, lane 1), revealed that the dephosphorylation of MtrA~P by the WT MtrB was higher than that of the mutant MtrB’, suggesting that the mutant SK had a lower phosphatase activity as compared to the WT SK (Fig 3B). This observation provides a plausible explanation for the sustained phosphotransfer from mutant MtrB to the RR, as seen in Fig 2B.

We also estimated the Pi released by the wild type and mutant SKs as an outcome of their phosphatase activity. Towards this, we performed Thin-Layer chromatography analysis and quantitated the amount of ^32^P labeled Pi released (from γ^32^P-ATP) during both the autophosphorylation and phosphotransfer reactions involving the WT or the mutant proteins at various time points. The autophosphorylation reaction for the wild type MtrB and mutant MtrB’ M517L protein generated similar amounts of Pi (S2A Fig in S1 File). However, the Pi release observed in the phosphotransfer reactions mediated by the mutant protein was lower than that in the WT protein. The difference was significant at 15 minutes and later time points of the phosphotransfer reaction. This experiment and phosphatase activity analysis (described above) suggested that the mutation impairs the phosphatase activity of the MtrB’ protein, thereby leading to sustained levels of MtrA~P (S2A and S2B Fig in S1 File), the effects of which can alter the downstream signaling of the cascade.

### Mutant MtrB’ protein enhances the binding of the RR MtrA to its target DNA

A direct consequence of the reduced phosphatase activity of the mutant MtrB’ protein would be an increase in the levels of phosphorylated RR MtrA~P in the cell, which will impact the expression of the downstream *mtrAB* TCS regulon. Given that MtrA is a DNA binding protein [32], we investigated its ability to bind to one of its target DNA loci corresponding to *oriC* [32], when phosphorylated through WT or mutant MtrB protein.

A 526 bp DNA fragment corresponding to the *oriC* region was end-labeled with ^32^P, and MtrA binding to it was evaluated by mobility shift experiments (EMSA). Initially, binding to unphosphorylated MtrA was tested to determine the amount for which the minimal binding is recorded, and 1.3 μM of MtrA did not retard the DNA movement (Fig 3C). Next, MtrA protein was incubated for various time durations with phosphorylated wild type or mutant MtrB’ proteins, followed by EMSA analysis with the labeled DNA in response to phosphorylation. In this experiment, we observed—(i) a substantial binding of MtrA to the DNA in response to phosphorylation (Fig 3D, early time points 2, 5 and 15 min), (ii) loss of DNA binding at later time points corresponding to the absence of phosphorylated MtrA due to phosphatase activity of SK (Fig 3D, late time points, 30 and 45 minutes) and (iii) a more significant reduction in the binding of MtrA generated through wild-type MtrB at 30 minutes or later phosphotransfer time points (Fig 3D), compared to the mutant protein, where the binding is better. We also confirmed that the SK by itself (unphosphorylated) does not bind to DNA (Fig 3D, right panel). Since phosphorylation of the RR enhances its DNA binding, this difference in binding can be attributed to higher phosphorylated MtrA at later time points due to the reduced phosphatase activity of the mutant MtrB’ SK. This finding suggests possible changes in downstream gene expression by the enhanced propensity of MtrA to bind to DNA upon the activation of the *mtrAB* TCS by the mutant MtrB protein.

### Overexpression of the mutant *mtrB’ in vivo* changes the expression levels of downstream genes in *Mycobacterium tuberculosis H37Ra*

The downstream genes regulated by the MtrAB operon have been reported previously [16, 33] and include genes that regulate cell division, reactivation from latency, and cell wall homeostasis. To study the changes in the downstream transcription profile resulting from the mutation, we used a mycobacterial expression vector pMV261, with a *hsp60* promoter to overexpress the wild type or the mutant *mtrB*’ *M517L* gene in the avirulent *M*. *tuberculosis H37Ra* strain. While the *H37Ra* strain is an avirulent isogenic variant of *M*. *tuberculosis* H37Rv, the prototypical virulent lab strain, there are no variations in the *mtrAB* loci between the two strains, making it a valuable system to study the basic impact of MtrB mutation. Previous studies have also used this system as a surrogate to express H37Rv proteins [34], examine the relation between TCSs and dormancy [35, 36] and virulence of mycobacteria [37].

**Fig 4:**
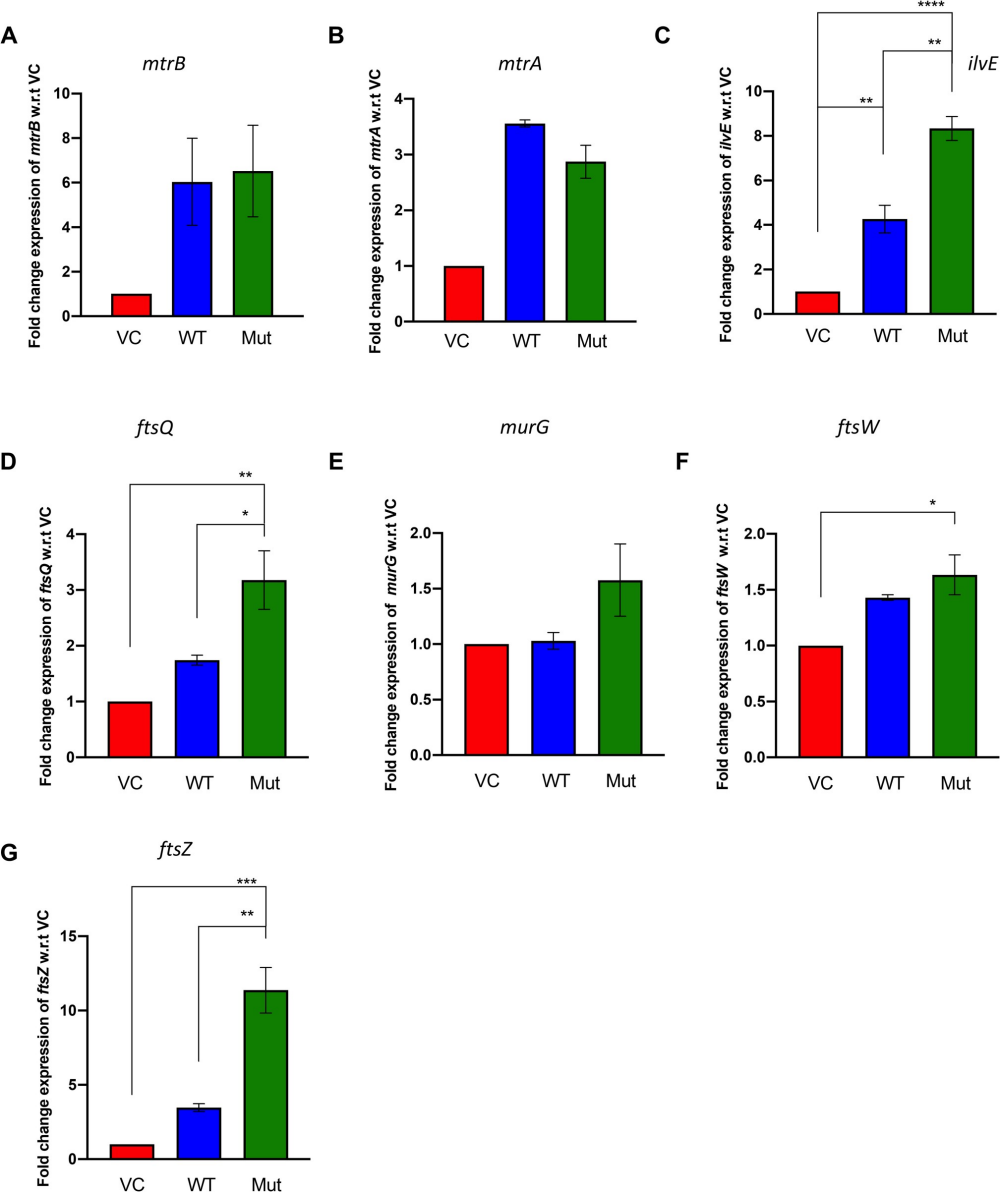
Assessment of the impact of MtrB mutation on downstream gene expression in *M*. *tuberculosis H37Ra*. **A-G.** Relative expression of mRNA levels in H37Ra strains over-expressing the wild-type or mutant *mtrB* cloned in the pMV261 vector and grown in Middlebrook 7H9 medium. The expression of the specific genes was normalized to the levels of 16S rRNA before calculating the fold change between test and vector control samples. The expression changes are shown for the **A.** *mtrB*; **B.** *mtrA*; **C.** *ilvE*; **D.** *ftsQ*; **E.** *murG*; **F.** *ftsW*; **G.** *ftsZ*. For all experiments, n = 3 biologically independent experiments. (*p* values; *≤ 0.05, **≤ 0.01, ***≤ 0.001, ****≤ 0.0001).

Furthermore, given that MtrAB system is also present in non-pathogenic Mycobacterial species and primarily regulates cell division, the use of the H37Ra strain was sufficient to address the role of this mutation. Keeping this focus, in the recombinant strains, we analyzed changes in the expression of downstream genes known to be regulated by MtrAB TCS such as *ilvE*, *murG*, *ftsQ*, *ftsW* and *ftsZ*, along with the changes in the levels of the *mtrA* and *mtrB* genes by qRT-PCR. The *ilvE* gene is involved in the metabolic regulation of the bacteria. The *murG*, *ftsQ*, *ftsW* and *ftsZ* genes are involved in regulating cell division and cell wall processes. The interaction between *ftsZ* and *mtrB* is known to be critical components of septa formation essential for the proper division of the cell [18]. As anticipated, the levels of *mtrB* were higher in the overexpression strains compared to the strain containing vector alone (Fig 4A). The expression levels of *mtrA* gene were slightly lower in the mutant when compared to the WT overexpression strain; however, this difference was not significant (Fig 4B). Previously, the expression of the selected genes was shown to be downregulated when the *mtrB* gene was knocked out [14]. In agreement with this, the levels of *ilvE*, *murG*, *ftsQ*, *ftsW* and *ftsZ* were higher in the strain overexpressing either the wild type or mutant *mtr*B. Their levels were significantly higher in the presence of mutant MtrB’ compared to the wild-type strain (Fig 4C–4G). Overall, we show that this mutation in the MtrB SK alters the expression of downstream genes of the MtrAB regulon *in vivo* and can affect the bacteria’s physiology.

### The strain expressing mutant MtrB’ show *a*ltered growth rates and altered membrane properties

**Fig 5:**
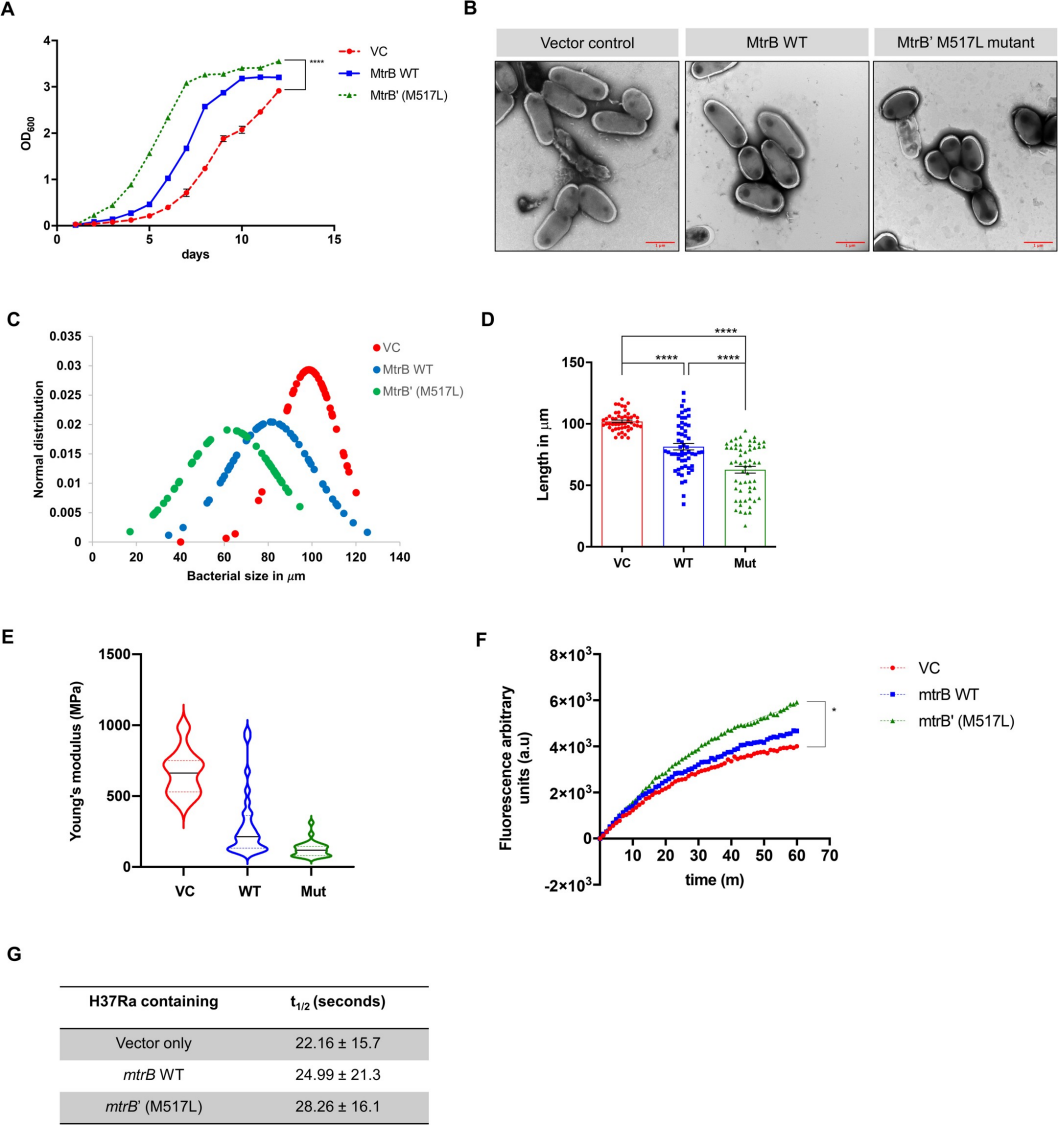
Analysis of growth rates and cell size of strains overexpressing wild-type or mutant MtrB. **A.** Growth curves of H37Ra strains overexpressing *mtrB* WT, *mtrB*’ (M517L) or the vector alone in 7H9 medium (containing 10% OADC, 0.5% glucose, 0.05% Tween80) (n = 3). **B.** Transmission electron microscopy analysis (TEM) of recombinant H37Ra strains. Representative micrographs of various strains as indicated. **C.** Normalized distribution of the bacterial cell length of various strains based on the EM images shown in Fig 5B. **D.** Average cell length of various strains calculated from EM images shown in Fig 5B. **E.** Representative topographical images of various strains (as indicated) by atomic force microscopy (AFM). The upper panel of images represent the peak force error maps for each sample and the lower panel of images show the height of each sample. **F.** Graph showing the nonlinear regression fit of the fluorescence curve obtained for the three strains vector control, *mtrB* WT and *mtrB’* M517L at an excitation-emission of 530–590 obtained after incubation with EtBr (statistical analysis performed only for the last time point of 60min). **G.** Membrane permeability calculated by the accumulation of EtBr over 60 minutes and represented as t_1/2_ in seconds at a concentration of 0.5 μM of EtBr. The slopes of fluorescence emission curve obtained in Fig 5G were used to determine the t_1/2_ value for membrane permeability. For all experiments, n = 3. (P values; *P ≤ 0.05, **P ≤ 0.01, ***P ≤ 0.001, ****P ≤ 0.0001).

Given that the MtrAB TCS target genes play a critical role in cell division and cell membrane synthesis [16], we next examined for changes in the growth rates and cell sizes of the recombinant strains containing wild-type or mutant *mtrB’*. Interestingly, in this study, we observed that the overexpression of either MtrB WT or mutant alone altered the growth rates, and strains carrying the MtrB’ mutant grew faster than both the wild-type or vector alone strains (Fig 5A). This could stem from the variation in the activity of MtrB available, which generates MtrA~P to regulate the expression of downstream genes involved in cell division. An increase in the expression of these genes (Fig 4C–4G) could, therefore, in turn, lead to increased growth rates of the strain.

Given that there were differences in growth rate, we next examined the strains for changes in the morphology of the bacilli. We observed that the bacilli expressing mutant MtrB’ protein were shorter and stubbier, lowering the average mean size of the population (Fig 5D). The strain expressing WT MtrB also had shorter bacilli compared to the vector alone, suggesting that just an increase in the levels of *mtrB* is sufficient to alter the cell size in the population. However, it was apparent that the mutation augments its effect, which was reinforced by electron microscopy analysis and quantitative length measurements followed by a normalized distribution of cell sizes (Fig 5B and 5C). Interestingly, when we analyzed for morphological differences in the colonies formed on plates of 7H11, we did not find any gross differences (S3 Fig in S1 File).

Since MtrB is also associated with the functional integrity of the cell membrane [14], we next used atomic force microscopy to analyze for differences in the mechanical properties of the cell membrane, such as the stiffness. We recorded a lower Young’s modulus, i.e., an increase in the rigidity of the membrane of the mutant and wild-type overexpression strain (Fig 5E and S4 Fig in S1 File) compared to the vector control. The significant increase in the membrane’s stiffness suggested a probable difference in the membrane permeability between these strains.

Given that the rigidity of the membrane is known to be directly proportional to its permeability [38, 39], we first analyzed if membrane permeability is altered over 60 minutes using the EtBr uptake assay [40]. We measured the accumulation of the EtBr in all three strains and found that the mutant shows a higher mean fluorescence intensity at 60 minutes than the vector control, indicative of a higher accumulation of EtBr in the cell (Fig 5F). Like what was recorded for cell length changes, the overexpression strain with wild-type MtrB also showed a higher fluorescence than the vector control. A nonlinear regression analysis fit on these curves revealed that the strain harboring the mutant *mtrB’* had the highest t_1/2_ value of 28.26 ± 16.1 min, compared to the strain overexpressing WT MtrB and vector control (Fig 5G). This could be due to changes in the ability to efflux EtBr from the system in the mutant strain because of a more rigid membrane. Previous reports also suggest the existence of variations in cell membrane composition among strains, shown by demonstrating differences in membrane staining [6]; therefore, a change in membrane composition may also be indicated by the increased membrane stiffness, retention, and permeability of the mutant.

## Discussion

Clues from comparative genomics revealing genetic variations across clinical strains of *M*. *tuberculosis* and H37Rv laboratory strain have provided insights into the evolution of mycobacterial strains. Recent reports describing higher levels of *mtrA* response regulator in XDR clinical isolates [20] have generated significant interest in the contribution of signaling proteins and mutations in mycobacterial growth and physiology. Here we report and characterize an SNP in MtrB SK present in 78% of the strains in the GMTV database. This SNP maps to the HATPase domain of the sensor kinase MtrB at the 517^th^ position changes a Methionine residue to Leucine and alters the biochemical activity of the sensor kinase. This change perturbs the MtrAB regulated signaling pathways impacting the rate of cell division and integrity of the cell membrane.

The spontaneous occurrence and maintenance of SNPs, insertions, and substitutions at low frequencies in the genome of *M*. *tuberculosis* have been deemed necessary, unlike in many other bacteria. The analysis of newly emerging and evolving strains of mycobacteria in different populations show that although the lineage and clades of these strains can be mapped back to a known and common ancestor, the behavior of the bacteria varies significantly due to the presence of SNPs in genes involved in drug resistance, virulence, and other regulatory genes [41]. Association studies have shown that mutations or SNPs in the RNA polymerase genes of rifampicin-resistant strains of Mycobacteria increase their fitness [42, 43]. While SNPs that affect drug resistance and virulence have now been established, their role in modifying the bacterium’s adaptive responses cannot be undermined [44].

The two-component signal transduction systems play an integral role in fine-tuning and coordinating cellular responses to numerous environmental cues. Thus, it is not surprising that deletion of two-component system *kdpDE* due to a frameshift at the C-terminal caused by an upstream SNP that increased the virulence of a Colombian clinical strain [45]. Through *in vitro* studies presented here, we demonstrate that a clinically prevalent SNP in the SK MtrB alters the core biochemical activities of the kinase. Given that the SK MtrB is known to be a non-essential gene in *M*. *tuberculosis* and crosstalks extensively with other TCS in M. tuberculosis [23], lower activity of the mutated SK in clinical strains strengthens its dispensable role and hints that its activation and regulatory activities of MtrAB TCS are governed mainly by its essential partner MtrA. Specifically, we report a change in the phosphatase activity associated with the MtrB protein, which impinges on the duration of activation of MtrA RR.

Interestingly, while many *M*. *tuberculosis* SK proteins have been shown to possess the ability to dephosphorylate their response regulator protein as a means to regulate the signaling event, such an activity has not been reported for MtrB SK so far. The identified SNP / mutation not only affected the autophosphorylation ability of the MtrB SK, but it also suppressed its dephosphorylation function such that the mutant kinase allowed sustained phosphorylation of its RR MtrA. A direct implication of this can be seen in the enhanced ability of the MtrA response regulator to bind to the DNA region corresponding to the *oriC* region, one of its regulatory targets.

Several studies have established a role for the *M*. *tuberculosis* MtrAB two-component system regulating cell division, survival within the host, and biofilm formation [16, 17]. Given the central role played by the MtrAB TCS in various mycobacterial species, overexpression of the wild type or the mutant *mtrB* gene (M517L) in *M*. *tuberculosis* H37Ra showed interesting phenotypes *in vivo*. As anticipated, these strains showed a variation in their growth rates, with the mutant showing the highest growth rate. Previous reports indicate that perturbing the *mtrA* gene levels and activity leads to the bacilli elongation [18]. In contrast, a knock-out of the *mtrB* gene causes the slowing down of growth and pole to pole extension of the bacilli [16, 18, 32, 46]. In agreement with these studies, we show that MtrB’ M517L expressing cells were smaller in length, implicating the M517L mutation in cell size and growth regulation. The observed variation in cell size can also be linked to changes in the mechanical properties of the cell membrane. In accordance, we report increased stiffness of the membrane with the overexpression of the *mtrB* gene. Membrane permeability across strains also varied, partly due to the interaction of MtrB with FtsI (penicillin-binding protein 3) and Wag31 (a cell wall synthesis protein) involved in the proper functioning of the cell membrane.

**Fig 6:**
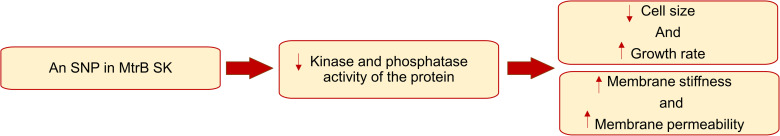
A schematic describing the downstream changes caused by the SNP in the MtrB SK via its altered signalling.

Overall, we show a high-frequency SNP in clinical strains of *M*. *tuberculosis* that affects functional activities of the MtrB sensor kinase, leading to changes in the cell size, permeability, and stiffness of the cell membrane in the mycobacterial cells (Fig 6). Similar mutation/s in the signaling proteins, if present in the clinical strains, can alter signaling and consequently change the bacilli’s physical properties, thus introducing a new layer of complexity to combat tuberculosis.

## Supporting information

S1 File Supplementary figures. (PDF)

S2 File Supplementary tables. (PDF)

S3 File Supplementary methods. (PDF)

S1 Raw images (PDF)
