# Supplementary material for: A high-frequency single nucleotide polymorphism in the MtrB sensor kinase in clinical strains of Mycobacterium tuberculosis alters its biochemical and physiological properties
Source: PLoS One. 2021 Sep 16;16(9):e0256664. doi: 10.1371/journal.pone.0256664 (PMC8445491; doi:10.1371/journal.pone.0256664)
Supplement: S1 File — (PDF) [file pone.0256664.s001.pdf]

Fig S1

A

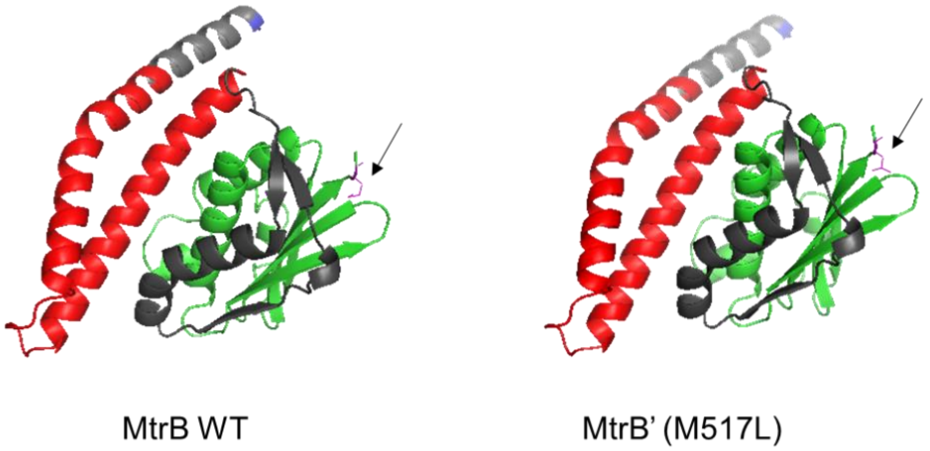

B

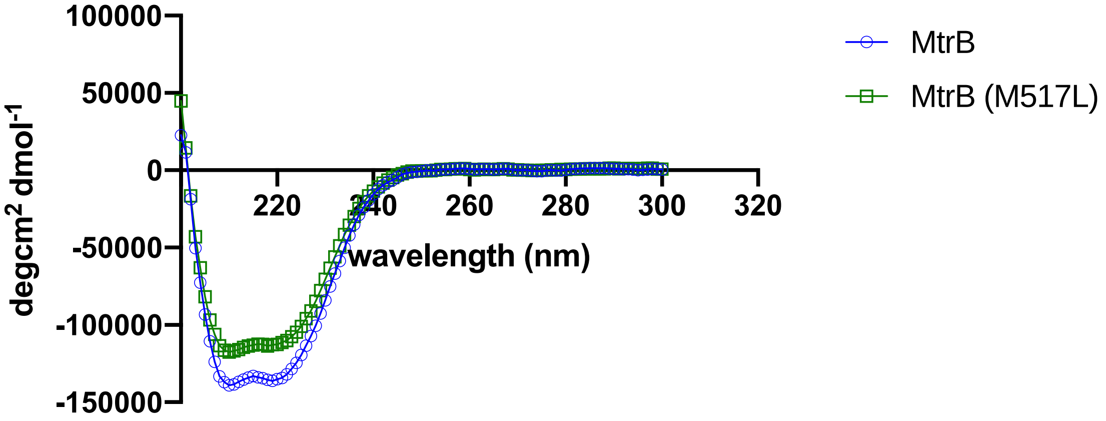

Fig S1. A. Predicted protein backbone ribbon structure model of the sensor kinase protein MtrB, using PHYRE [1]. Left, wildtype MtrB protein and right, mutant MtrB protein (MtrB’ M517L). The kinase and HATPase domain are shown. The mutation at the 517th position from a methionine to leucine is marked by an arrow. **B.** Circular dichroism analysis of MtrB WT and the mutant MtrB’ (M517L) proteins. Circular dichroism of the proteins was evaluated using a Jasco Spectropolarimeter. Ellipticity was measured from 200 nm to 300 nm in 1 mm pathlength and 50nm/ sec scanning speed. The data were analyzed with K2D3 Dichroweb software.

Fig S2

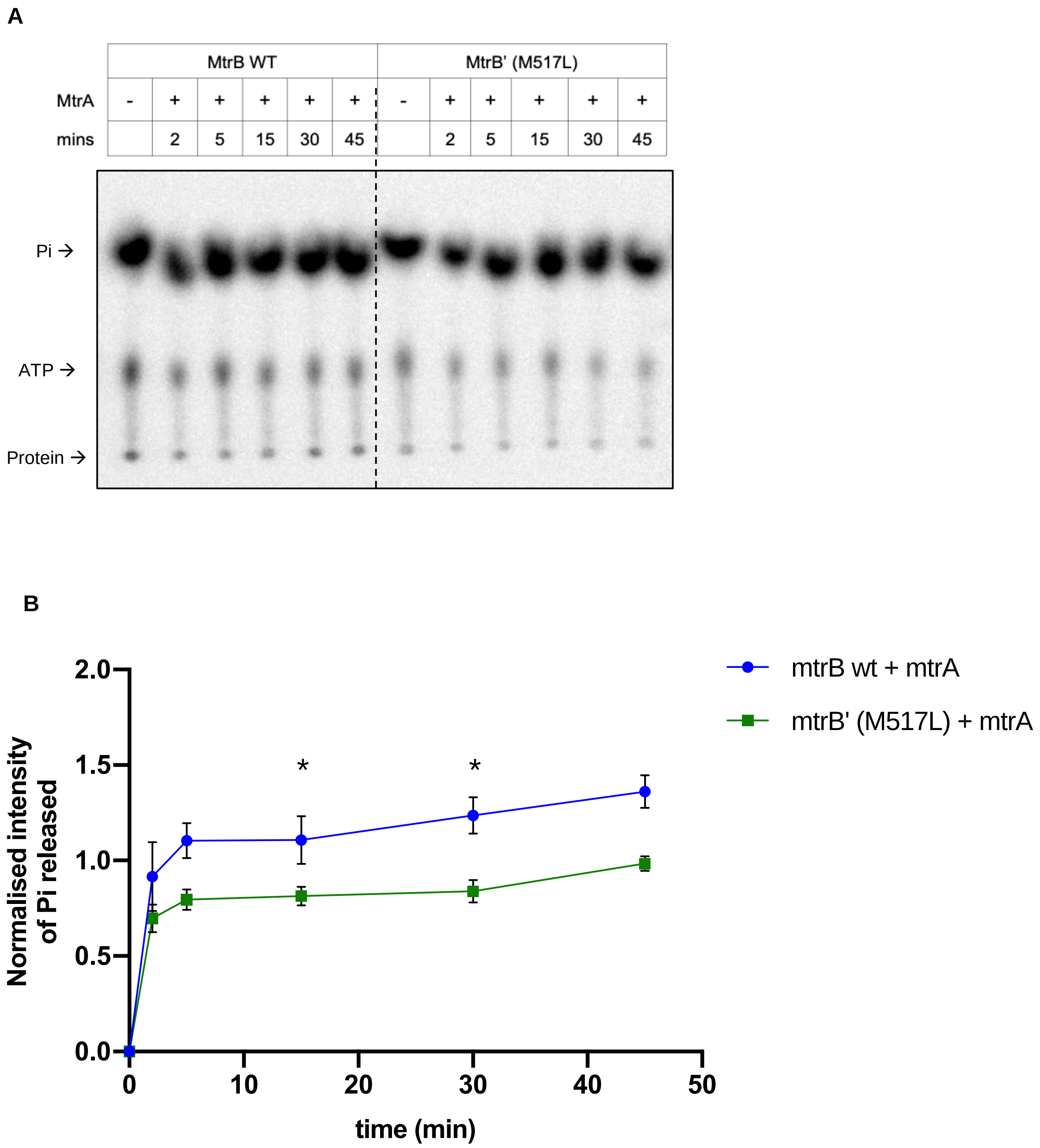

Fig S2. A. Image of TLC analysis of phosphotransfer time course to determine the effect of the mutation on net ATP hydrolysis. TLC was performed as described in the materials and methods section. **B.** Quantitative measurement of the amount of inorganic phosphate (Pi) generated by the WT or the mutated protein in the presence of RR MtrA at various time points. The Pi generated by the SK alone post 2 hours of autophosphorylation was taken as 1 and the respective timepoints of the phosphotransfer reaction were normalized to it (n=3). The P value was calculated based on the amount of Pi generated in the presence of the RR (MtrA) with respect to SK~P alone (p values; *≤ 0.05, **≤ 0.01).

Fig S3

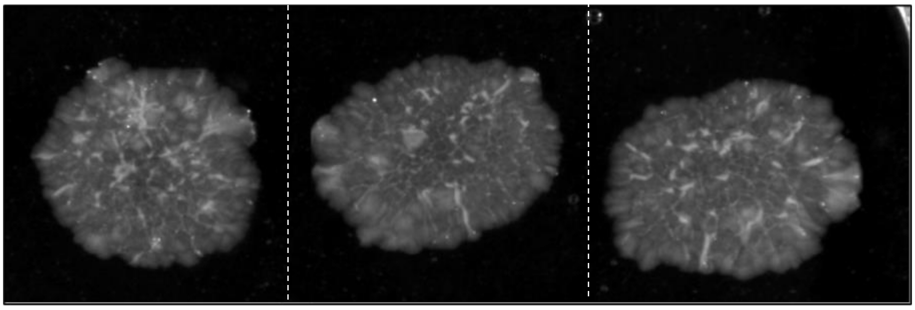

Vector Control *mtrB* WT *mtrB*' (M517L)

Fig S3. Colony morphology of the three strains of *H37Ra* carrying the recombinant plasmids, basic vector containing no insert, vector control; expressing *mtrB WT;* or *mtrB' (M517L)* (mtrB mutant copy) as indicated. The strains were grown on 7H11 media plate containing kan^{+} for 14 days and imaged.

Fig S4

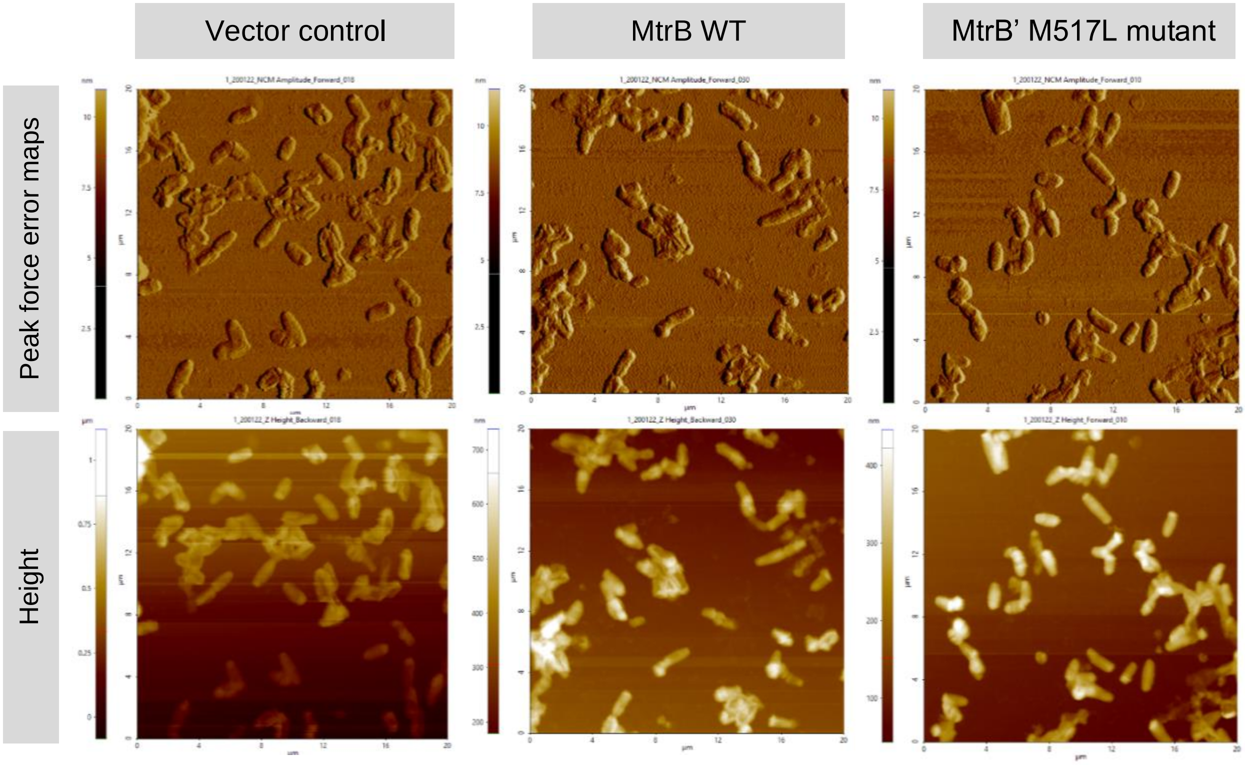

Fig S4. Representative topographical images of various strains (as indicated) by atomic force microscopy (AFM). The upper panel of images represent the peak force error maps for each sample and the lower panel of images show the height of each sample.

References:

1. Kelly SM, Jess TJ, Price NC. How to study proteins by circular dichroism. Biochim Biophys Acta - Proteins Proteomics. 2005;1751: 119–139. doi:10.1016/j.bbapap.2005.06.005
