# Supplementary material for: A high-frequency single nucleotide polymorphism in the MtrB sensor kinase in clinical strains of Mycobacterium tuberculosis alters its biochemical and physiological properties
Source: PLoS One. 2021 Sep 16;16(9):e0256664. doi: 10.1371/journal.pone.0256664 (PMC8445491; doi:10.1371/journal.pone.0256664)
Supplement: S2 File — (PDF) [file pone.0256664.s002.pdf]

## SUPPLEMENTARY TABLE

### 1) List of primers used in this study.

| Primers | Sequence | Reference |
|---|---|---|
| *mtrB* M517L fwd | 5’ GCTGACGCTTCCGCTGGTGCGCGGCCAC 3’ | This study |
| *mtrB* M517L rvs | 5’ CGCGCACCAGCGGAAGCGTCAGCCG 3’ | |
| *mtrB* pMV fwd | 5’ GTACGAATTCAATGGCCAGGCTGGCGGTGTC 3’ | This study |
| *mtrB* pMV rvs | 5’ GATCAAGCTTCAACCGCTCCACTCCGCG 3’ | |
| *oriC* prom fwd | 5’ CATCCGTCAGCGCTCC 3’ | This study |
| *oriC* prom rvs | 5’ TTGCGCCCTTTCACCTCACGATG 3’ | |
| *mtrA rt* fwd | 5’ GAACAGGTATGGGGTTACCG 3’ | [1] |
| *mtrA rt* rvs | 5’ TTGTACCCCACTCCTCGAAC 3’ | |
| *mtrB rt* fwd | 5’ TTGAGTCGAGCCGTAGCTGT 3’ | This study |
| *mtrB rt* rvs | 5’ ATGTCGAGGACACGATTGGT 3’ | |
| *dnaA rt* fwd | 5’ ACTCATCGCCAGCAGTATCG 3’ | This study |
| *dnaA rt* rvs | 5’ ATTTGCATGGTGTTGGCGTC 3’ | |
| *16s* rRNA rt fwd | 5’ ACGCGAAGAACCTTACCTGG 3’ | [2] |
| *16s* rRNA rt rvs | 5’ CACCTTCCTCCGAGTTGACC 3’ | |

### 2) List of Plasmids used in this study

| Plasmids | Source | Reference |
|---|---|---|
| Rv3245C, MtrB cloned in pPROEx-HT vector | Lab collection | [3] |
| Rv3246C, MtrA cloned in pPROEx-HT vector | Lab collection | |
| pProEx-HT MtrB’ M517L | Lab collection | This study |
| pMV261 | Lab collection | [4] |
| pMV261-*mtrB* | Lab collection | This study |
| pMV261-*mtrB’* M517L | Lab collection | This study |

### References


1. Goyal R, Das AK, Singh R, Singh PK, Korpole S, Sarkar D. Phosphorylation of PhoP protein plays direct regulatory role in lipid biosynthesis of Mycobacterium tuberculosis. J Biol Chem. 2011;286: 45197–45208. doi:10.1074/jbc.M111.307447

2. Singh KK, Bhardwaj N, Sankhe GD, Udaykumar N, Singh R, Malhotra V, et al. Acetylation of Response Regulator Proteins, TcrX and MtrA in M. tuberculosis Tunes their Phosphotransfer Ability and Modulates Two-Component Signaling Crosstalk. J Mol Biol. 2019;431: 777–793. doi:10.1016/j.jmb.2019.01.004

3. Agrawal R, Pandey A, Rajankar MP, Dixit NM, Saini DK. The two-component signalling networks of Mycobacterium tuberculosis display extensive cross-talk in vitro. Biochem J. 2015;469: 121–134. doi:10.1042/BJ20150268

4. C. K. Stover, V. F. de la Cruz, T. R. Fuerst, J. E. Burlein, L. A. Benson, L. T. Bennett, G. P. Bansal, J. F. Young, M. H. Lee, G. F. Hatfull, S. B. Snapper, R. G. Barletta WRJJ& BRB. New use of BCG for recombinant vaccines. Nature. 1991;354: 56–58.
