# Supplementary material for: A high-frequency single nucleotide polymorphism in the MtrB sensor kinase in clinical strains of Mycobacterium tuberculosis alters its biochemical and physiological properties
Source: PLoS One. 2021 Sep 16;16(9):e0256664. doi: 10.1371/journal.pone.0256664 (PMC8445491; doi:10.1371/journal.pone.0256664)
Supplement: S3 File — (PDF) [file pone.0256664.s003.pdf]

Supplementary methods

Expression and purification of recombinant proteins. In brief, the expression plasmids for the sensor kinase and response regulator proteins were transformed in to *E. coli* cells and grown at 37°C in 200 ml of Terrific broth (TB) to an OD_{600}>1.0. They were then induced with IPTG (0.1-1.0 mM). For the expression of proteins, the culture was further grown for 15-20 h at 10-13°C. The soluble 6×His tagged proteins were purified using Ni^{+2}-NTA after cells were harvested by centrifugation, as described previously [1]. The pellets obtained were resuspended in native lysis buffer (50mM of Tris Cl, pH 8, 300mM NaCl, 10% glycerol) containing 1mM PMSF and 1 mM benzamidine and sonicated on ice for 5-10 minutes at 25 % amplitude (pulse on 3s, pulse off 2s). The lysate was centrifuged at 12000 rpm for 30 mins at 4°C and the supernatant containing the protein was passed through a Ni^{2+}-NTA column pre-equilibrated with native lysis buffer for about 120 minutes with intermittent shaking at 4°C for 15 min. The unbound proteins were discarded as flow through and the column was washed with wash buffer A (25 mM Tris.Cl pH 8.0, 500 mM NaCl, 25 mM imidazole, 10% glycerol) and wash buffer B (25 mM Tris.Cl pH 8.0, 500 mM NaCl, 50 mM imidazole, 10% glycerol). The elution buffer (25 mM Tris.Cl pH 8.0, 500 mM NaCl, 250 mM imidazole, 10% glycerol) was used to elute out bound protein.

Dialysis and storage of purified proteins. Bradford's assay was used to determine the yield of the protein and the eluted fractions of the proteins with high yields were pooled together and dialyzed against dialysis buffer I (50 mM Tris.Cl, pH 8.0, 50 mM NaCl, 1 mM DTT, 10% glycerol) for 6-12 hrs and followed by dialysis against II or storage buffer (50 mM Tris.Cl, pH 8.0, 50 mM NaCl, 0.1 mM DTT, 50% glycerol) overnight. The concentration of the purified proteins after dialysis was determined by

Bradford assay using BSA as standard and the purity of the proteins was checked on SDS-PAGE and stored at -20°C.

RNA extraction and quantitative gene expression analysis. Cultures of *M. tuberculosis H37Ra* containing either the pMV261 empty vector backbone alone or either the wildtype or mutant *mtrB* construct were grown exponentially to an OD_{600} of 0.8 and harvested. The pellets were resuspended in QIAzol Lysis reagent (Qiagen, Germany), mixed with zirconia silica beads and disrupted using a mini bead beater following which RNA was precipitated using ethanol. The RNA obtained was treated with DNaseI and 500 ng of the total RNA was reverse transcribed using random hexamers and iScript reverse transcriptase (Qiagen, Germany) as per manufacturer’s protocol. Gene specific qRT-PCR was performed using DyNAmo Color Flash SYBR Green qPCR Kit (Thermo Scientific, USA) in Roto-GeneQ cycler (Qiagen, Germany). The calculated threshold cycle (Ct) value for each gene was normalized to 16S rRNA followed by that of the gene of interest in the strain containing vector only to determine fold change. The expression analyses were performed using three independent biological replicates.

Circular dichroism spectroscopy. MtrB WT and MtrB’ M517L proteins were subjected to circular dichroism spectroscopy using JASCO J-810 Spectropolarimeter. Spectra for 100 µg of the SK protein (in 1×PBS) was analyzed between wavelength 190 to 300 nm to record protein secondary structures. The K2D3 software was used to analyse the plots.

Cell permeability assays. Ethidium bromide (EtBr) uptake and accumulation was measured by fluorescence intensity, as previously described [2] with minor modifications. Briefly, actively growing mid-log phase cultures were washed thrice

with PBS and the OD_{600} to 0.1 and 100µl of each culture was aliquoted into each well in a 96well plate. These were stained with 5µg of EtBr (Sigma) and readings were taken 544/590 nm excitation emission on the plate reader M10000 (TECAN) at every 60seconds for an hour. All data was normalised to time zero and OD of each well and performed in triplicates. The $t_{1/2}$ was calculated by nonlinear regression fit of the fluorescence curves obtained over an hour and the significance was calculated at the last time point of 60 minutes.

References:

1. Agrawal R, Pandey A, Rajankar MP, Dixit NM, Saini DK. The two-component signalling networks of Mycobacterium tuberculosis display extensive cross-talk in vitro. Biochem J. 2015;469: 121–134. doi:10.1042/BJ20150268

2. Campodónico VL, Rifat D, Chuang YM, Ioerger TR, Karakousis PC. Altered Mycobacterium tuberculosis cell wall metabolism and physiology associated with RpoB mutation H526D. Front Microbiol. 2018;9: 494. doi:10.3389/fmicb.2018.00494
