# Supplementary material for: A high-frequency single nucleotide polymorphism in the MtrB sensor kinase in clinical strains of Mycobacterium tuberculosis alters its biochemical and physiological properties
Source: PLoS One. 2021 Sep 16;16(9):e0256664. doi: 10.1371/journal.pone.0256664 (PMC8445491; doi:10.1371/journal.pone.0256664)
Supplement: S1 Raw images — (PDF) [file pone.0256664.s004.pdf]

Figure 2A

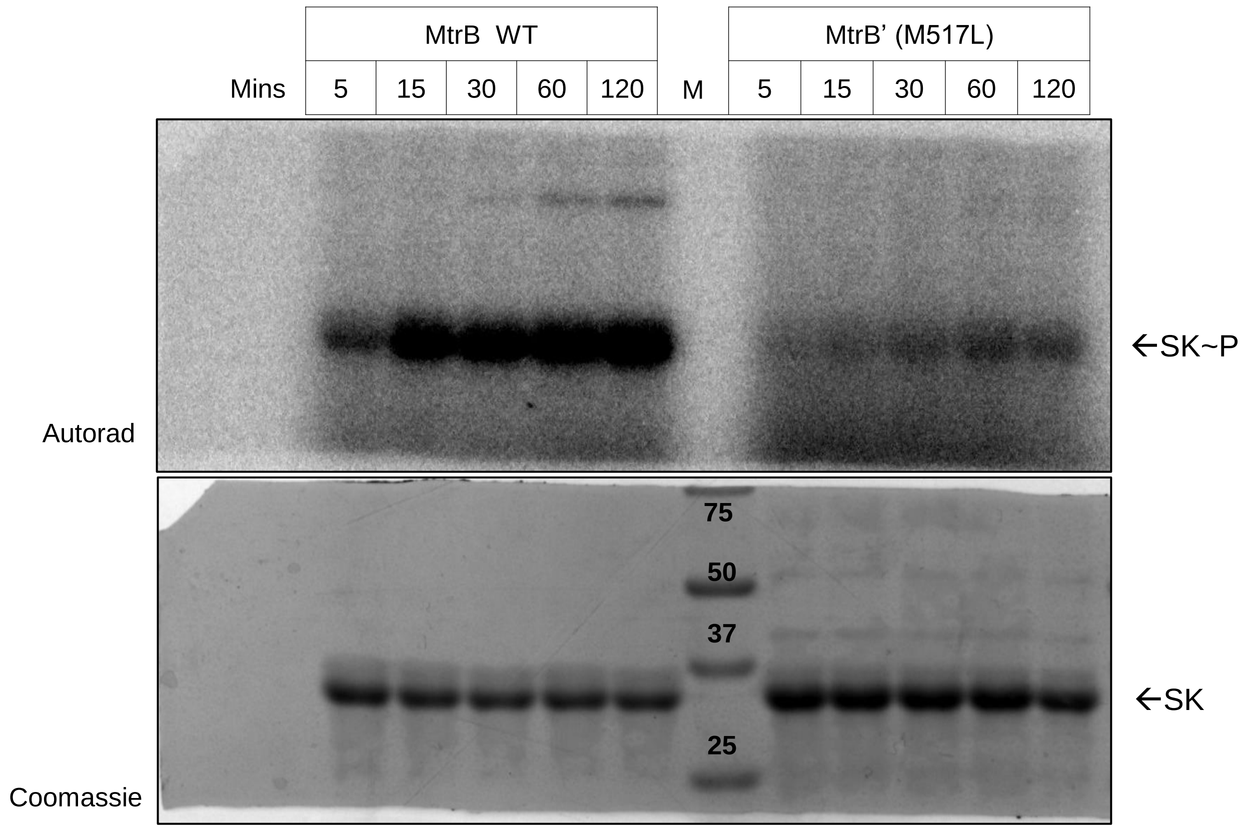

Figure 2C

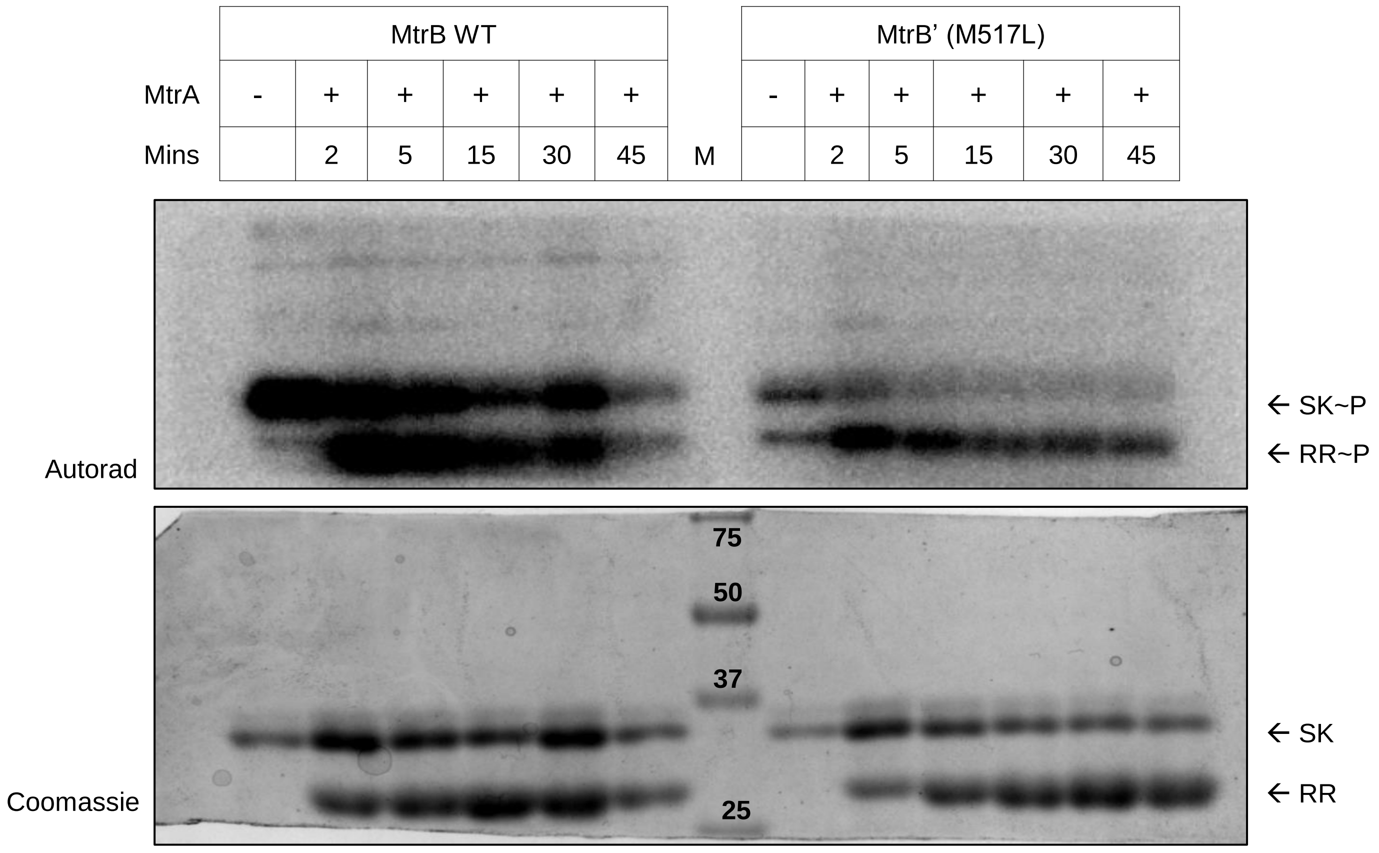

Figure 3A

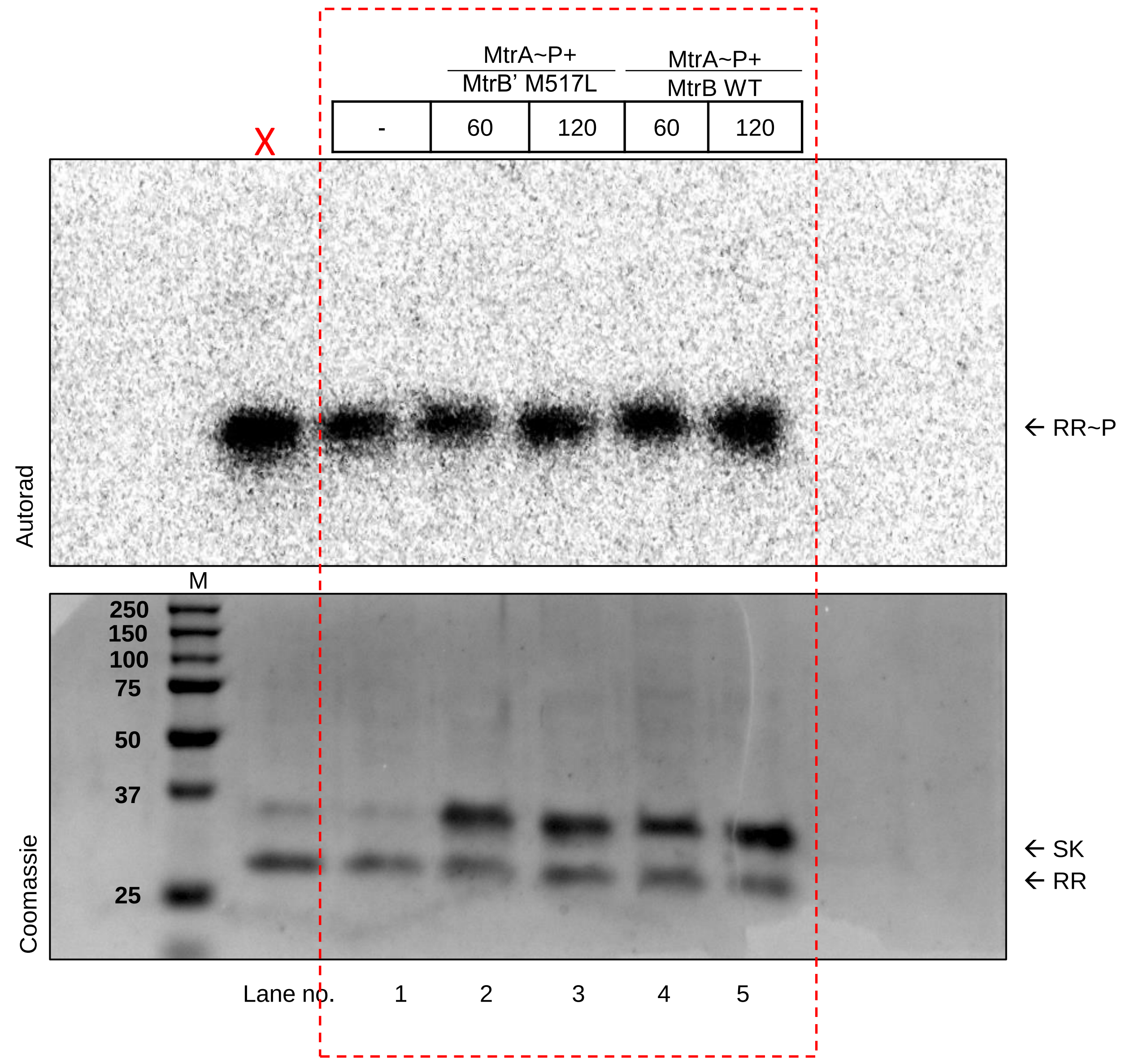

Lanes marked in Red are shown in the Figure 3A

Figure 3C

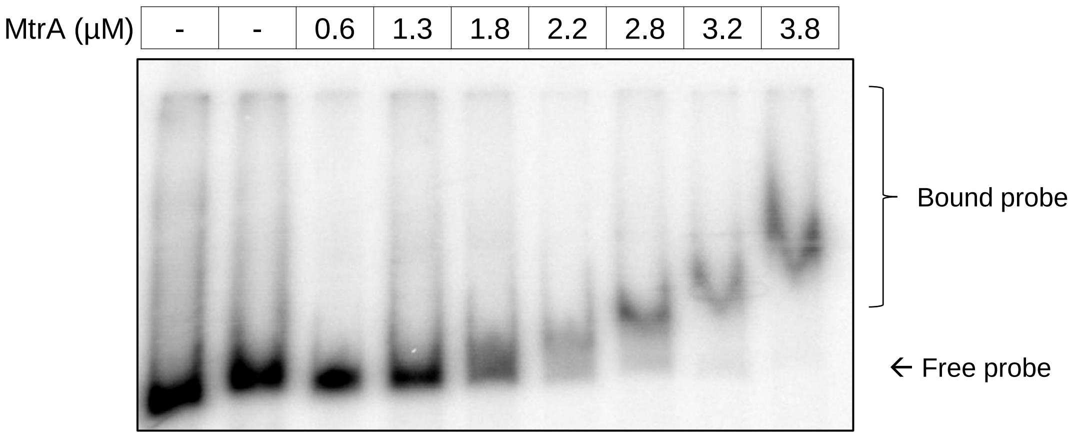

Figure 3D

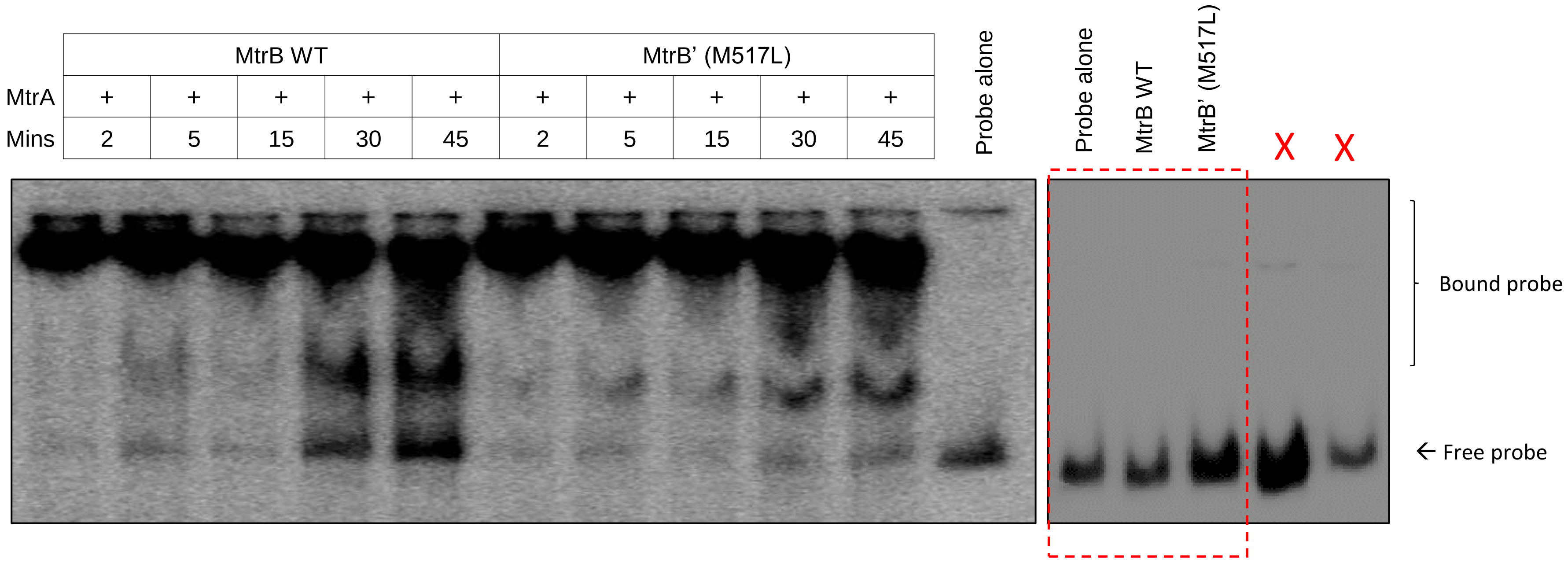

Lanes marked in Red are shown in the Figure 3D
